# Live-imaging studies reveal how microclots and the associated inflammatory response enhance cancer cell extravasation

**DOI:** 10.1242/jcs.261225

**Published:** 2023-09-28

**Authors:** Juma Ward, Paul Martin

**Affiliations:** School of Biochemistry, Biomedical Sciences Building, University of Bristol, University Walk, Bristol BS8 1TD, UK

**Keywords:** Cancer, Inflammation, Zebrafish, Coagulation

## Abstract

Previous clinical studies and work in mouse models have indicated that platelets and microclots might enable the recruitment of immune cells to the pre-metastatic cancer niche, leading to efficacious extravasation of cancer cells through the vessel wall. Here, we investigated the interaction between platelets, endothelial cells, inflammatory cells, and engrafted human and zebrafish cancer cells by live-imaging studies in translucent zebrafish larvae, and show how clotting (and clot resolution) act as foci and as triggers for extravasation. Fluorescent tagging in each lineage revealed their dynamic behaviour and potential roles in these events, and we tested function by genetic and drug knockdown of the contributing players. Morpholino knockdown of fibrinogen subunit α (*fga*) and warfarin treatment to inhibit clotting both abrogated extravasation of cancer cells. The inflammatory phenotype appeared fundamental, and we show that forcing a pro-inflammatory, *tnfa*-positive phenotype is inhibitory to extravasation of cancer cells.

## Teaser

**Highlighted Article:** Live imaging in zebrafish larvae reveals that fibrin and platelets in the pre-metastatic niche can recruit pro-metastatic innate immune cells and drive cancer metastasis.

## INTRODUCTION

Clinical observations for more than a century have indicated that thrombosis is associated with cancer progression and metastatic spread. Indeed, Armand Trousseau predicted his own death through cancer malignancy after experiencing episodes of deep vein thrombosis (Metharom et al., 2019; Trousseau, 1865). More recent experimental studies have revealed that this link is, in part, because many cancers release pro-thrombotic factors (Tawil et al., 2021; Yu et al., 2005), but platelets and fibrin might also act to protect blood-borne cancer cells from shear forces (Egan et al., 2014) and immune attack (Gorelik et al., 1984). Furthermore, microclots might function as a favoured pre-metastatic niche for immune cell recruitment and cancer cell extravasation (Gil-Bernabé et al., 2012). To date, these experimental studies have been largely undertaken in mouse models in which the opaque tissues make live-imaging investigations difficult.

Large cohort population health studies have further cemented the bidirectional relationship between coagulation and metastatic cancer. Several single nucleotide polymorphisms have been associated with hyperactivation of platelets and the coagulation cascade, leading to an increased risk of venous thromboembolism (Tavares et al., 2020a). Many of the factors identified in these genome-wide association studies are also linked to a more rapid disease progression and metastasis in cancer patients (Li et al., 2021; Tavares et al., 2020b; Werfel et al., 2020). Additionally, long-term treatment with warfarin, a common anti-coagulant, appears to lower the incidence of cancer (Haaland et al., 2017).

Given this plethora of evidence linking clotting and cancer metastasis, we used the translucency of zebrafish larvae to live image the dynamic relationship between cancer cells, endothelial cells, platelets (thrombocytes in fish; Khandekar et al., 2012) and immune cells. We investigated spontaneous cancer cell extravasation episodes and those at sites of microclots, in standard conditions and after pharmacological or genetic inhibition of clot formation.

## RESULTS AND DISCUSSION

**Fig. 1.**
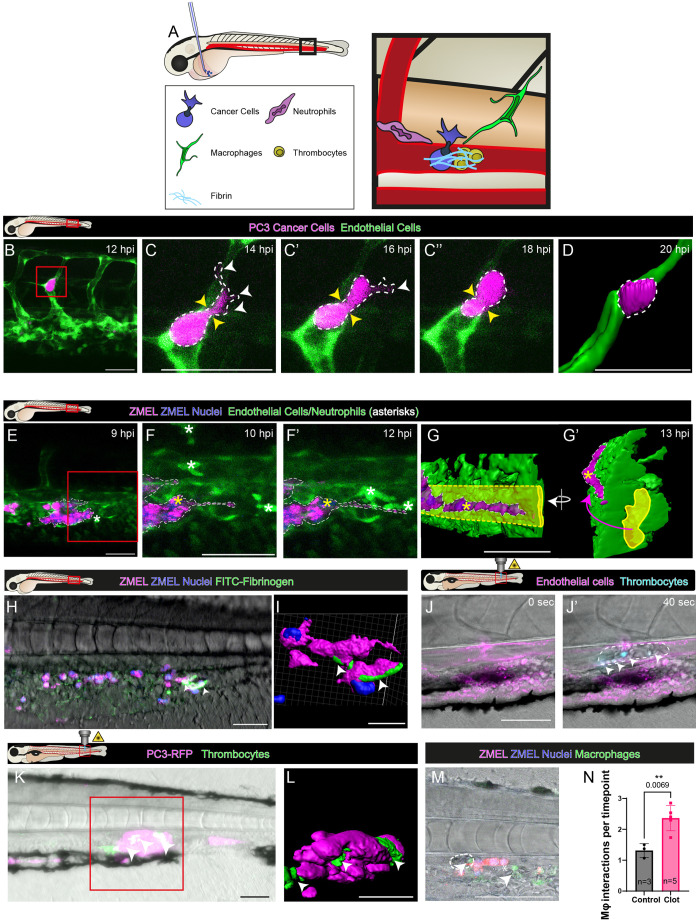
**Microclot formation at the pre-metastatic niche is essential for cancer cell extravasation.** (A) Schematic representation of our zebrafish larval extravasation model. Macrophages and neutrophils interact with grafted cancer cells arrested at the site of a microclot. (B) Still image from a live confocal movie (Movie 1) as a human prostate cancer cell extravasated from an intersegmental vessel of a 2 dpf zebrafish larva. The box indicates the area magnified from the movie shown in C. (C–C″) Time-lapse images of extravasation. White arrowheads indicate invadopodial extensions outside of the vessel and yellow arrowheads indicate pinch points as the cell squeezes through the vessel wall. Dotted lines represent the boundaries of the cell. (D) 3D Imaris rendering of the same cancer cell just after extravasation. (E) Confocal image of zebrafish melanoma (ZMEL) cells within a vessel of a 2 dpf larva. The box indicates the area magnified from Movie 2 shown in F,F′. (F,F′) The same cells as in E at later timepoints. Neutrophils (white asterisks) interact directly with a cancer cell as it extravasates (Movie 2). The nucleus of the extravasating cell is marked with a yellow asterisk. (G,G′) Imaris-rendered projections of the same ZMEL cell just after exiting the vessel (yellow) (Movie 3). (H) Flank of a 2 dpf larvae, injected with FITC-labelled human fibrinogen prior to the grafting of ZMEL cancer cells. Fibres of fibrin(ogen) (arrowheads) form at the surface of cancer cells. (I) Imaris rendering of fibres (arrowheads) on the surface of cancer cells. (J,J′) Formation of a laser-induced clot (dashed line) within the dorsal aorta of a 3 dpf zebrafish larva. Arrowheads indicate mature thrombocytes. (K) Human cancer cells interacting with activated thrombocytes of a microclot. (L) Imaris rendering of the boxed area in K to reveal intimate cancer cell–thrombocyte interactions. Arrowheads in K,L indicate activated thrombocytes. (M) At a microclot (dashed line), macrophages (arrowheads) interact with ZMEL cancer cells (asterisks). (N) Quantification of macrophage (Mϕ) interactions with cancer cells in the presence or absence of a microclot (two-tailed unpaired Student's *t*-test, two independent repeats). Data show the mean±s.d. Images are representative of ≥3 independent experiments. Scale bars: 50 µm.

To model cancer cell extravasation in zebrafish larvae, we used a grafting model of metastatic cancer (Chen et al., 2021; Drabsch et al., 2017). Human or zebrafish cancer cells were injected directly into the blood vessels of larvae, and live imaging was performed to assess the metastatic potential of circulating tumour cells (CTCs) and their dependency on platelets, fibrin and innate immune cell interactions (Fig. 1A).

### Both human and zebrafish cancer cells injected into the larval zebrafish bloodstream associate with fibrin(ogen) prior to extravasation through the vessel wall

Live imaging of injected zebrafish melanoma (ZMEL) and human prostate cancer (PC3) cells as they spontaneously extravasated through vessel walls of intersegmental vessels revealed significant morphological contortions as they probed and then squeezed their way through the vessel wall (Fig. 1B–G). The human cancer cells are larger than zebrafish cells (20 μm versus 10 μm in diameter) and extravasated considerably slower than the zebrafish cells, taking up to 8 h (versus 0.5–3 h) from the first invadopodial extension to fully exiting the vasculature. We observed innate immune cells interacting with cancer cells throughout the extravasation process (Fig. 1F,F′).

Previous studies have suggested that the association of platelets and fibrin with CTCs might influence metastatic behaviour and disease progression (Anvari et al., 2021; Gil-Bernabé et al., 2012; Labelle et al., 2014). To investigate whether CTCs associate with fibrin(ogen) before they extravasate through the vessel wall, we live imaged 2-day-post-fertilisation (dpf) larvae injected with FITC-labelled human fibrinogen (FITC–hFibrinogen). Within minutes of injecting ZMEL cancer cells, FITC–hFibrinogen fibres formed at the surface of CTCs and persisted for more than 12 h (Fig. 1H,I). This association suggests that CTCs catalyse the local conversion of fibrinogen into fibrin.

### Neutrophils and macrophages associate with cancer cells at laser-activated microclot pre-metastatic sites

An ultraviolet (UV) ablation laser was used to lightly damage the lining of the dorsal aorta of 3 dpf larvae, and the resulting microclots were allowed to occlude the vessel and stabilise before injection of cancer cells (Fig. 1J,J′). Injected cancer cells halted at the site of occlusion, where they become intimately associated with thrombocytes (Fig. 1K,L). These capture sites rapidly recruited neutrophils and macrophages, and thus became the foci for inflammatory cell–cancer cell interactions, to a considerably greater extent than sites where cancer cells were more distant from the clot (Fig. 1M,N).

Our own previous studies have shown that neutrophils and macrophages are both recruited to sites of pre-neoplastic cell initiation and provide these cells with trophic support (Feng et al., 2012, 2010), and, at the pre-metastatic niche, they might play similarly nurturing roles for cancer cells.

### CLEM reveals close interactions between human cancer cells and host innate immune cells

**Fig. 2.**
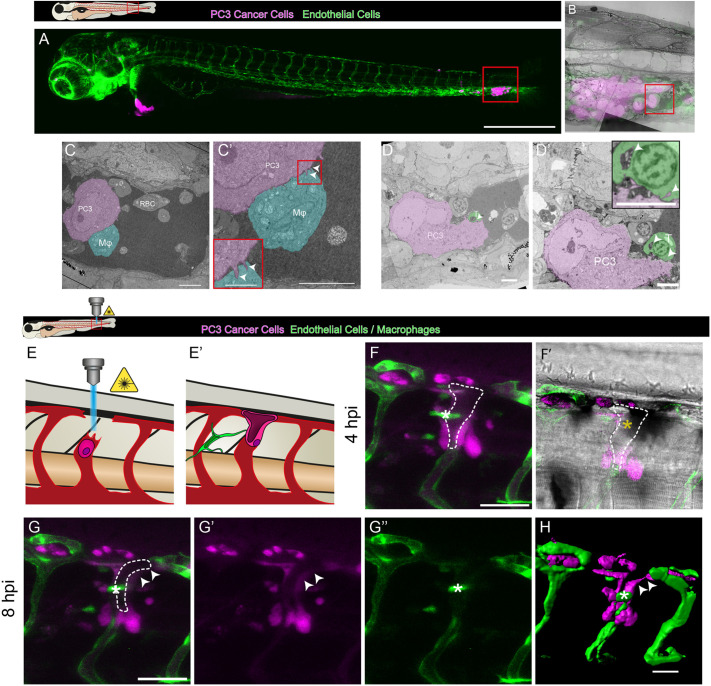
**CLEM views of cancer cells interacting closely with host innate immune cells and vasculature of the pre-metastatic niche.** (A) View of human prostate cancer cells (magenta) post injection into the vasculature (green) of a 3 dpf larval zebrafish. The box indicates the higher-magnification image shown in B. (B) Overlay of light microscopy and transmission electron microscopy (TEM) images of this region. The red box in B indicates the higher-magnification image shown in C. (C,C′) Direct interactions between a macrophage (Mϕ, cyan) and a cancer cell (PC3, magenta), including interdigitation (arrowheads, C′) between the membranes of these cells. RBC, red blood cell. (D) TEM image of a human cancer cell (PC3, magenta) interacting closely with a zebrafish thrombocyte (T, green). (D′) Higher-magnification image of the same cancer cell shown in D, 5 µm deeper. The inset shows close membrane contacts with possible membrane exchange (arrowheads). (E,E′) Schematic of cancer cell integration into the larval vasculature. (F,F′) Fluorescence (F) and brightfield (F′) still images from a time-lapse movie of a human cancer cell (magenta) integrating into a vessel (green) (Movie 4). The dashed line indicates the cancer lumen. (G–G″) Multi- and split-channel still images from the same time-lapse. (H) Imaris rendering of the same cancer cell undergoing vascular integration. Arrowheads in G,G′,H indicate cancer cell extensions into the vasculature. White asterisks in F–H indicate vessel-associated macrophages, whereas yellow asterisks indicate blood cells within the lumen. Images are representative of ≥3 independent experiments (A–D′), and a single experiment (F–H). Scale bars: 500 µm (A); 10 µm (C,C′); 2.5 µm (C′ inset); 5 µm (D,D′); 50 µm (F–H).

We set out to gain further insight into the nature of interactions between grafted cancer cells and host cells using correlative light and electron microscopy (CLEM). We first identified the time and location at which cancer cells had aggregated (Fig. 2A), and then fixed these larvae and processed them for electron microscopy, enabling us to capture macrophage–cancer cell interactions at subcellular detail (Fig. 2B). We saw direct membrane–membrane contacts and interdigitations between the membrane of cancer cells and macrophages (Fig. 2C,C′). Direct interactions of this kind have previously been reported in *in vitro* studies to induce M2-like, pro-tumoural phenotypes in macrophages and, simultaneously, to increase tumour cell proliferation (Komohara et al., 2012).

### At the microclot niche, cancer cells take up platelet material and contribute to the pre-metastatic niche vasculature

Our CLEM studies show that thrombocytes, too, interact directly with cancer cells and might undergo exchange of material (Fig. 2D,D′). Recent studies investigating *in vitro* co-culture of cancer cell lines with primary human platelets have shown active uptake of platelet material into cancer cells (Martins Castanheira et al., 2022). Furthermore, tumour-educated platelets might act as a cancer biomarker, indicating the presence and progression of the disease in patients (Best et al., 2017).

Vasculogenic mimicry is a phenomenon wherein cancer cells contribute to the tumour vasculature (Fernández-Cortés et al., 2019; Wei et al., 2021). On occasions after laser ablating an intersegmental vessel close to arrested cancer cells, we observed human cancer cells migrating to bridge the gap between the remaining undamaged vessels and integrating with the local vasculature (Fig. 2E–H). During this process, we saw macrophages maintaining long and close interactions with these vessel-associated cancer cells and, as macrophages have been shown to act as drivers of vasculogenic mimicry (Liu et al., 2022), perhaps mirroring the nurturing roles of macrophages seen during wound angiogenesis (Gurevich et al., 2018). We speculate that recruitment of macrophages to cancer cells in the presence of activated thrombocytes indicates a role for thrombosis in the vasculogenic mimicry process.

### Warfarin blocks macrophage interactions with cancer cells, and knockdown of macrophages leads to reduced extravasation

**Fig. 3.**
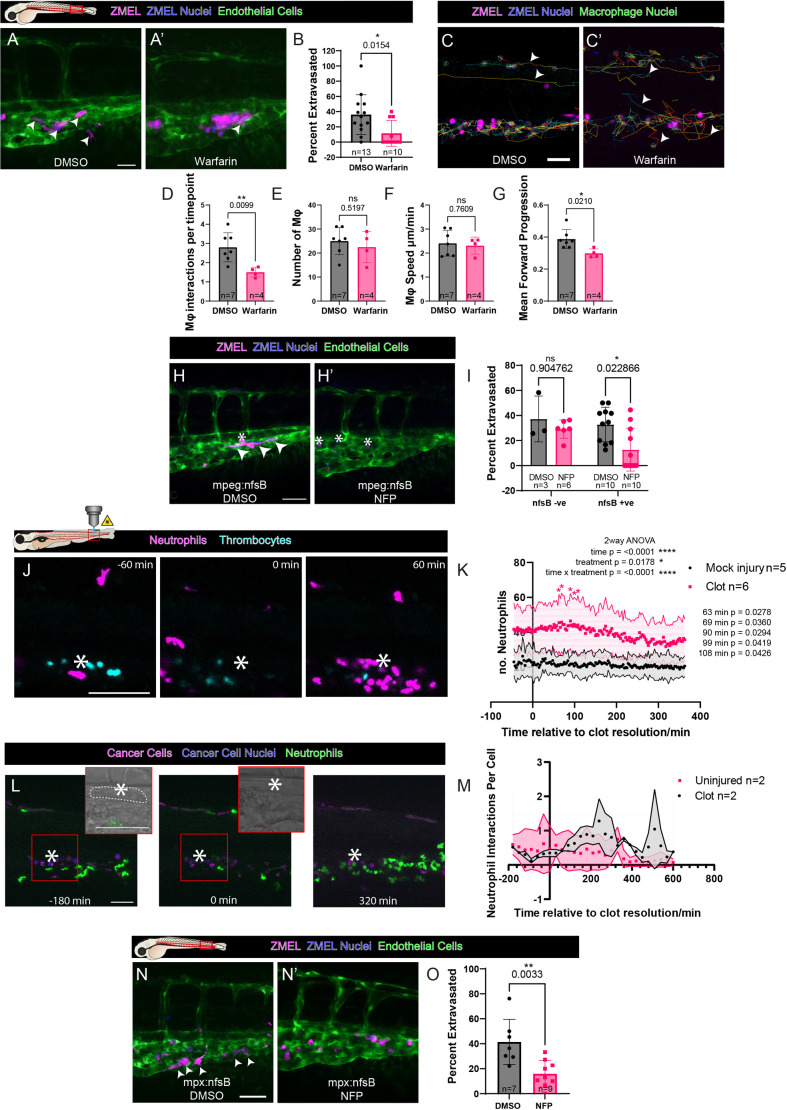
**Microclot association with cancer cells leads to innate immune cell recruitment and aids extravasation.** (A,A′) Confocal microscopy flank view of control versus warfarin-treated 3 dpf larvae injected with ZMEL cancer cells. Arrowheads indicate cancer cells that have exited vessels. (B) Quantification of the percentage of cancer cells that have extravasated tail vessels in control versus warfarin-treated larvae (Mann–Whitney *U*-test). (C,C′) Tracks of macrophage movements in zebrafish larvae injected with ZMEL cancer cells over a 14 h period. Arrowheads indicate macrophage tracks that are not local to cancer cells. (D–G) Quantification of macrophage interactions with individual cancer cells (D), total macrophages detected (E), macrophage speed (F) and macrophage directionality (G) in control versus warfarin-treated larvae (two-tailed unpaired *t-*tests). (H,H′) Confocal images of macrophages expressing *NfsB* in control versus nifurpirinol (NFP)-treated larvae after intravascular injection of ZMEL cancer cells. Extravasated cancer cells are marked with arrowheads; intravascular cells are marked with asterisks. (I) Percentage of cancer cells extravasated in the tail regions of *NfsB*^+^ or *NfsB*^−^ larvae after treatment with NFP (multiple Mann–Whitney *U*-tests with Holm–Šídák corrections). (J) Still images from a time-lapse movie of neutrophil recruitment to the site of a laser-induced clot, extending until after the clot has resolved (Movie 5). Asterisks indicate the location of the laser injury. (K) The number of neutrophils at various timepoints and two-way ANOVA analysis. The timepoint 0 is standardised to the time of clot resolution. Asterisks indicate significance using post hoc Šídák's multiple comparisons test between treatment groups. (L) Time-course imaging of neutrophils recruited to ZMEL cancer cells captured at the site of a laser-induced microclot (white dashed line). Insets show brightfield images of the boxed regions. Asterisks indicate the location of the laser injury. (M) Quantification of number of neutrophils interacting with cancer cells captured within a microclot over time (representative of a single experiment). Shaded areas in K,M represent s.d. (N,N′) Representative images of NFP-treated larvae with *NfsB-*expressing neutrophils after injection of ZMEL cancer cells (magenta with blue nuclei). Arrowheads indicate extravasated cancer cells. (O) Percentage of cancer cells extravasated after neutrophil ablation (two-tailed unpaired *t*-test). Data show the mean±s.d. and are representative of ≥3 independent repeats, unless otherwise stated. Scale bars: 50 µm. ns, not significant.

To test the impact of clotting on cancer cell extravasation, we blocked coagulation by treating larvae with warfarin for 24 h prior to injection of cancer cells. This treatment was sufficient to abrogate the occlusion of vessels by thrombocytes and prevented the formation of fibrin(ogen) fibres on the surface of cancer cells. The flanks of these larvae were imaged 24 h post injection (hpi) and the numbers of extravasated cancer cells were significantly reduced in comparison to those in DMSO-treated control larvae (Fig. 3A,B).

Platelets and clots have previously been shown to increase innate immune cell recruitment to the pre-metastatic niche (Gil-Bernabé et al., 2012; Labelle et al., 2014). Immune cells and cancer cells were tracked using TrackMate (Tinevez et al., 2017), and interactions between these cells were considered ‘close’ if immune cells approached within 30 μm of CTCs. Indeed, in warfarin-treated larvae, we saw a significant reduction in the numbers of macrophages recruited to CTCs, with only half as many ‘close’ interactions between macrophages and cancer cells observed (Fig. 3C,D). Macrophage numbers and speed within the tail were unaltered (Fig. 3E,F), but the mean directionality of macrophage movement was reduced, compared to those of DMSO-treated larvae (Fig. 3G).

To investigate whether the loss of macrophage–CTC interactions might directly cause the reduction in extravasating cancer cells seen in warfarin-treated larvae, we next used a macrophage ablation line [Tg(*mpeg*:Galf4FF); Tg(*UAS*:NfsB-mCherry)]. In this line, a nitroreductase enzyme (*NfsB*), specifically expressed in macrophages, allows the conversion of the pro-drug nifurpirinol (NFP) to cytotoxic products, causing cell-specific ablation. 10 µM NFP was added to the larval medium 24 h prior to cancer cell injection. The subsequent loss of macrophages resulted in a reduction in the numbers of extravasating cancer cells to about half of the numbers in controls (Fig. 3H,I), suggesting that direct macrophage interactions with cancer cells might be important, although not essential, for cancer cell extravasation. To ensure that NFP itself did not directly influence cancer cell extravasation, *NfsB*-negative sibling larvae were also imaged and showed no significant difference in extravasation when treated with NFP.

### Neutrophil recruitment peaks at the onset and subsequently upon resolution of a microclot, and knockdown of neutrophils reduces extravasation

As with macrophages, we observed an increase in neutrophil interactions with cancer cells in larvae with a laser-induced clot over larvae without such a microclot (Fig. 3J,K). Curiously, there was an even larger influx of neutrophils immediately following the resolution of the clot, which might reflect a model of ‘ischaemic reperfusion’ as blood flow recommenced (Fig. 3K). A two-way ANOVA confirmed a transient peaking of neutrophils shortly after the resolution of a clot and this was also the peak time for neutrophil–cancer interactions (Fig. 3L,M). This might have clinical relevance as ischemic reperfusion injury has been shown to be a major risk factor in cancer recurrence in hepatocellular carcinoma patients following surgery (Grąt et al., 2018).

To test whether neutrophil–cancer cell interactions might play a key role in cancer cell extravasation, we ablated neutrophils by neutrophil-specific *NfsB* expression under the *mpx* promoter. Indeed, we observed a significant reduction in cancer cell extravasation when treated with NFP (Fig. 3N,O), indicating that neutrophils, like macrophages, might be important in this key step in metastatic spread.

### Loss of fibrin causes an increase in pro-inflammatory macrophages

**Fig. 4.**
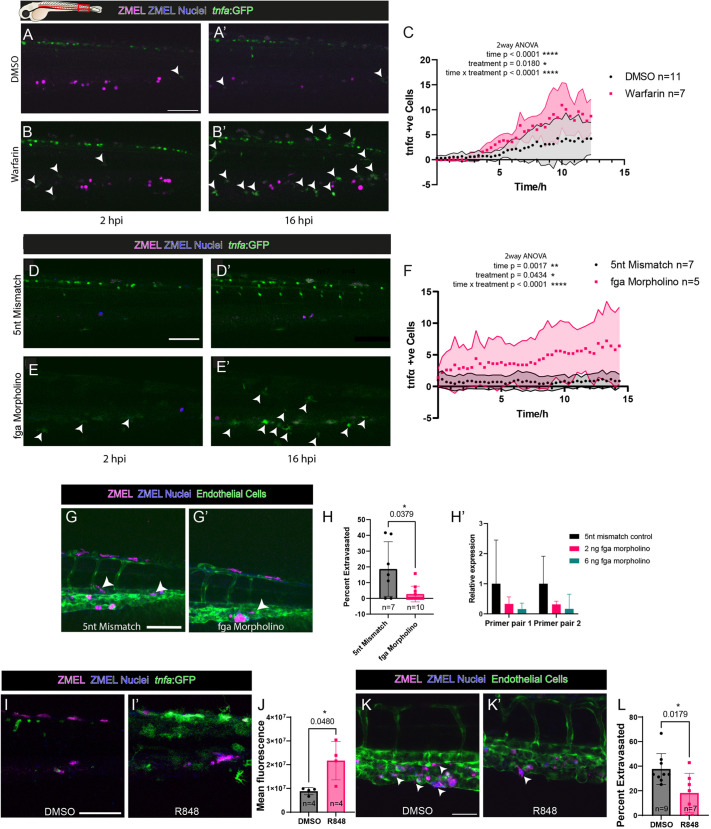
**Loss of fibrin drives a switch to a pro-inflammatory macrophage phenotype, which inhibits extravasation.** (A–B′) Confocal imaging of the larval flank to show recruitment of *tnfa*-positive macrophages (green, arrowheads) after intravascular injection of ZMEL cancer cells treated with either warfarin or DMSO. (C) Change in numbers of *tnfa*-positive cells over time (two-way ANOVA). (D–E′) Confocal imaging to reveal *tnfa*-positive macrophages (green, arrowheads) within the tails of *fga* morpholino-treated 2 dpf larvae injected with ZMEL cancer cells. (F) Change in numbers of *tnfa*-positive cells over time (two-way ANOVA). Shaded areas in C,F represent s.d. (G,G′) Confocal images of control (G) and active morpholino-treated (G′) larvae after injection of cancer cells. Arrowheads indicate extravasated cancer cells. (H) Quantification of the percentage of cancer cells extravasated after morpholino treatment (Mann–Whitney *U*-test). (H′) qPCR quantification showing loss of the *fga* transcript with morpholino treatment, representative of three technical repeats from a single experiment. (I,I′) Representative images of *tnfa*-positive cells (green) in ZMEL cancer cells injected in resiquimod (R848)-treated larvae. (J) Quantification of *tnfa* expression levels in control versus R848-treated larvae (two-tailed unpaired *t*-test), representative of two independent repeats. (K,K′) Representative images of ZMEL cancer cells within (arrowheads, K) and extravasated from (arrowhead, K′) the vasculature of 3 dpf larvae 24 hpi after R848 treatment. (L) Percentage of extravasated cancer cells seen in the tails of R848-treated larvae (Mann–Whitney *U*-test). Data show the mean±s.d. and are representative of ≥3 independent repeats, unless otherwise stated. Scale bars: 100 µm (A,D,G,I); 50 µm (K).

Prior studies have reported that different macrophage polarisation states within the pre-metastatic niche might greatly influence the metastatic outcomes for a cancer (Park et al., 2020; Zhou et al., 2022). To determine the polarisation status of macrophages, we used a *tnfa* reporter line [Tg(*tnfa*:GFP); Tg(*mpeg*:mCherry)] (Marjoram et al., 2015), which is used as an indicator of pro-inflammatory state (López-Cuevas et al., 2021; Nguyen-Chi et al., 2015). Live imaging of cancer-grafted larvae revealed an increase in the numbers of *tnfa*-expressing macrophages in the vicinity of cancer cells when treated with warfarin, compared to those in DMSO control treatment (Fig. 4A–C). Interestingly, the initial levels of *tnfa*-expressing cells were similar with very few *tnfa*-positive cells in either condition, despite larvae being treated with warfarin for 24 h prior to cancer cell injection. This suggests that warfarin alone does not act as a pro-inflammatory agent but acts synergistically, in the presence of cancer cells, to drive an M1-like response in macrophages.

Recently, fibrin has been shown to drive the pro-resolution, M2-like phenotype in macrophages and to dampen the effects of pro-inflammatory signals that might drive them towards the pro-inflammatory, M1-like phenotype (Borrego et al., 2022; Hsieh et al., 2017; Tanaka et al., 2019; Zhang et al., 2020). To test whether loss of fibrin might drive an increase in pro-inflammatory phenotypes and an increase of *tnfa* expression, we used a splice-site-disrupting morpholino against fibrinogen subunit α (*fga*) injected at the one-cell stage, which has been shown to lead to a loss of functional fibrinogen in zebrafish larvae, causing clotting defects (Vilar et al., 2021; Vo et al., 2013). Morpholino knockdown of *fga* led to an increase in *tnfa*-expressing cells (Fig. 4D–F). This morpholino treatment led to a significant reduction in cancer cell extravasation (Fig. 4G,H), suggesting an association between macrophage phenotype and extravasation efficacy of cancer cells, and further implicating a pro-extravasation role for fibrin within the pre-metastatic niche.

### Other treatments that switch macrophages to a pro-inflammatory phenotype reduce cancer cell extravasation

To test causality more directly, we investigated whether an increase in pro-inflammatory macrophages was sufficient to cause a reduction in extravasation when coagulation was unaltered. Here, we used a Toll-like receptor 7/8 (TLR7/TLR8) agonist, resiquimod (R848), which has been shown to drive inflammation in zebrafish (Muire et al., 2017) and inhibit cancer metastasis in mouse models (Anfray et al., 2021; Zhou et al., 2022). Intravascular injection of R848 led to a dramatic increase in cells expressing *tnfa* 24 h post treatment (Fig. 4I,J) and, when these larvae were injected with cancer cells, we saw a significant reduction in the percentage of extravasating cancer cells (Fig. 4K,L).

In summary, our live imaging and CLEM data show a clear indication of how microclots might lead to extravasation of blood-borne cancer cells through their interactions at these foci with fibrin, platelets and innate immune cells. We show genetic and pharmacological evidence that knockdown of fibrin or innate immune cells retards cancer cell extravasation, thereby hinting at potential targets for therapeutic inhibition of clot-mediated cancer cell extravasation. Further studies will reveal more mechanistic insights concerning the molecular signals underpinning each of these cell–cell interactions, and we suggest that zebrafish are an ideal model to observe these conversations and potentially to screen drugs that might halt this step in cancer metastatic invasion.

Previous studies have indicated important roles for both platelets and fibrin within the pre-metastatic niche (Gil-Bernabé et al., 2012; Wang et al., 2022), linked to their cancer cell protective capacity (Egan et al., 2014; Gorelik et al., 1984) and to signalling to the endothelium and immune cells (Schumacher et al., 2013; Labelle et al., 2014). Here, we suggest a mechanism by which fibrin might drive metastasis by recruiting innate immune cells but preventing their pro-inflammatory polarisation. This speculation is bolstered by prior works showing that fibrin has an anti-inflammatory influence on macrophages (Borrego et al., 2022; Hsieh et al., 2017; Zhang et al., 2020).

## MATERIALS AND METHODS

### Zebrafish lines and maintenance

All animal experiments were conducted in accordance with the Animals Scientific Procedures Act 1986 (ASPA), the Animal Welfare Act 2006 and were ethically approved by the University of Bristol Animal Welfare and Ethical Review Body (AWERB).

We used transgenic lines for fluorescence labelling of cells of interest, including Tg(*itga2b*:GFP) for thrombocytes (Lin et al., 2005), Tg(*fli*:GFP) for endothelial cells of vessels (Lawson and Weinstein, 2002), Tg(*cfms*:GFP) (Dee et al., 2016), Tg(*mpeg1*:nls-Clover) for macrophages (Bernut et al., 2019), Tg(*lyz*:DSRed) for neutrophils (Hall et al., 2007) and Tg(*tnfa*:GFP) for indication of pro-inflammatory status (Marjoram et al., 2015). Additional lines were crossed to allow selective cell ablation: Tg(*mpx*:Gal4FF) (Robertson et al., 2014), Tg(*mpeg1*:Gal4FF) (Ellett et al., 2011) and Tg(UAS:*NfsB-mCherry*) (Davison et al., 2007).

### Engraftment of tumour cells

The human cancer cell lines PC3-RFP and MDA-MB231-RFP (gifts from Catherine Nobes, University of Bristol) were maintained at 37°C, 5% CO_2_ and 10% humidity in Dulbecco's modified Eagle medium (DMEM) with sodium bicarbonate and phenol red (Sigma-Aldrich), supplemented with 10% fetal bovine serum (FBS; Sigma-Aldrich), 1% L-glutamine and 1% penicillin/streptomycin.

Zebrafish ZMEL-1 cancer cells (gift from Richard White, University of Oxford) (Heilmann et al., 2015) were maintained at 28°C, 5% CO_2_ and 10% humidity, in DMEM/F12 (Thermo Fisher Scientific) supplemented with 10% FBS, 1% penicillin/streptomycin, 1% L-glutamate, 1% amphotericin B (Biotechne) and 1% non-essential amino acids. ZMEL cells were fluorescently labelled immediately prior to harvesting. Adherent cells were first washed with PBS, before incubating with 5 µl/ml Vybrant-DiI (Thermo Fisher Scientific) and 1.6 µM Hoechst 33342 (Thermo Fisher Scientific) for 15 min at 28°C.

Cancer cells were harvested and suspended at 5×10^7^ cells/ml in PBS with 2% polyvinylpyrrolidone (Thermo Fisher Scientific). Zebrafish larvae at either 2 or 3 dpf were anaesthetised with 0.003% tricaine (Thermo Fisher Scientific) and laid out on a 1% agarose pad, and roughly 100 cancer cells were delivered into the vasculature via the duct of Cuvier by microinjection. Larvae injected with human cancer cells were then maintained at 33°C, whereas larvae injected with ZMEL were maintained at 28°C.

### Drug treatments

Drugs were added to the larval media 24 h prior to engraftment of cancer cells and embryos were dechorionated at the time of drug addition, where needed. Warfarin (Merck) was used at 50 mg/ml to prevent coagulation. Nifurpirinol (NFP) (Merck) was used at 10 µM to ablate innate immune cells. 1 nl resiquimod (R848) (Merck) at 10 nM in ≥99.9% DMSO was injected into the duct of Cuvier 24 h prior to cancer cell injections.

### FITC fibrinogen treatment

FITC-labelled human fibrinogen (Stratech) in PBS was injected into the Duct of Cuvier 30 min prior to cancer cell injections.

### Morpholinos

Zebrafish embryos were injected with either *fga* morpholino (5′-GCATTATATCACTCACCAATGCAGA3-′) or a 5-nt mismatch morpholino (5′-GCTTAATATGACTCACGAATCCAGA-3′) (GeneTools). The injection mix was made up to 2 ng/nl in Tris buffer, pH 7.4. 1 nl of injection mix was microinjected into the yolk within 10 min of fertilisation. Transcript levels were assessed by quantitative PCR (qPCR) using SYBR Green (Thermo Fisher Scientific). Primer pairs 1 (5′-ACCGATGATGACTGGGGAAG-3′ and 5′-ACTTCATCGGCAGAGCGATA-3′) and 2 (5′-CACATTCACTGCTCTGCCTG-3′ and 5′-TCATCATCGGTACACCCTGG-3′) were used to generate *fga* cDNA.

### Imaging

For confocal imaging, anaesthetised zebrafish larvae were mounted in 1% low-melting point agarose (Sigma-Aldrich) in glass-bottomed multi-well dishes (ibidi) and imaged under a Leica TCS SP8 AOBS confocal laser scanning inverted microscope.

For CLEM studies, confocal images were acquired as described and then larvae were freed from agarose before fixation in 4% paraformaldehyde (Thermo Fisher Scientific), 2.5% glutaraldehyde (Thermo Fisher Scientific) and 0.1 M sodium cacodylate (Thermo Fisher Scientific) buffer overnight at 4°C. Larvae were then washed in 0.1 M sodium cacodylate (three times for 10 min) before being placed in a secondary fixative containing 1% OsO_4_, 1.5% potassium ferrocyanide and 0.1 M sodium cacodylate for 2 h at 4°C. They were again washed in 0.1 M sodium cacodylate (three times for 10 min) before being placed in an automatic tissue processor (Leica EM TP), mounting with EPON and polymerisation. 70 nm sections were cut with a UC6 Leica ultramicrotome with Diamond Knife (Diatome), and were collected onto pioloform-coated copper slot grids (Agar Scientific). These sections were then imaged using a Tecnai 12 BioTwin Spirit TEM (tungsten filament, 120 kV) and a FEI Eagle 4k×4k CCD camera.

### Image analysis

Extravasated cells were quantified using anonymised data. From collected three-dimensional (3D) stacks, cells were assessed for their position relative to the vessel lumen and endothelial layer. Cells that were transendothelial and, therefore, partially extravasated were counted as extravasated.

Cellular interactions were determined using live imaging. TrackMate (Tinevez et al., 2017) was used to identify the coordinates of cells over time. For 3D reconstructions, imaging data were processed using Imaris (version 7.6.5).

### Statistical analysis

GraphPad Prism was used for statistical tests and generation of graphs. Normality was assessed using the Shapiro–Wilk test. For individual comparisons, two-tailed unpaired Student's *t*-test or Mann–Whitney *U*-test was used. Additionally, Fisher's exact tests were used to compare the percentages of extravasated cells. For cases where multiple comparisons were made, two-way ANOVA or Kruskal–Wallis tests were used, with post hoc multiple comparison tests. *P*=values lower than 0.05 were considered statistically significant.

## Supplementary Material



10.1242/jcs.261225_sup1 Supplementary information
