# Supplementary material for: Live-imaging studies reveal how microclots and the associated inflammatory response enhance cancer cell extravasation
Source: J Cell Sci. 2023 Sep 28;136(18):jcs261225. doi: 10.1242/jcs.261225 (PMC10561694; doi:10.1242/jcs.261225)
Supplement: Supplementary information [file joces-136-261225-s1.pdf]

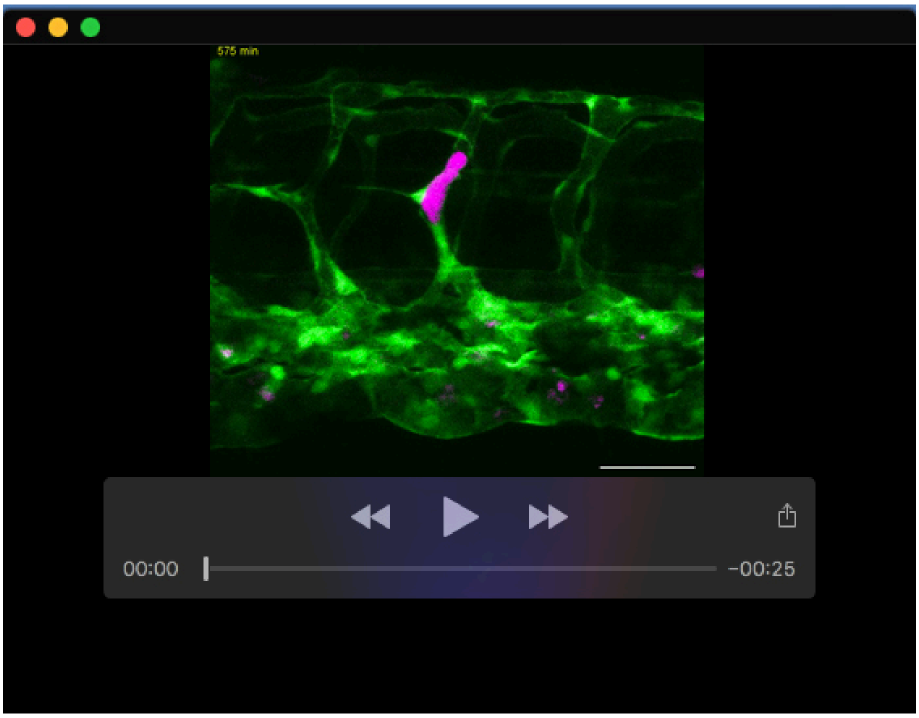


**Movie 1.** Timelapse of a human MDA-MB-231 cancer cell (magenta) extravasating from an intersegmental vessel (green) of a 2 dpf larva. Scale bar = 50 µm.

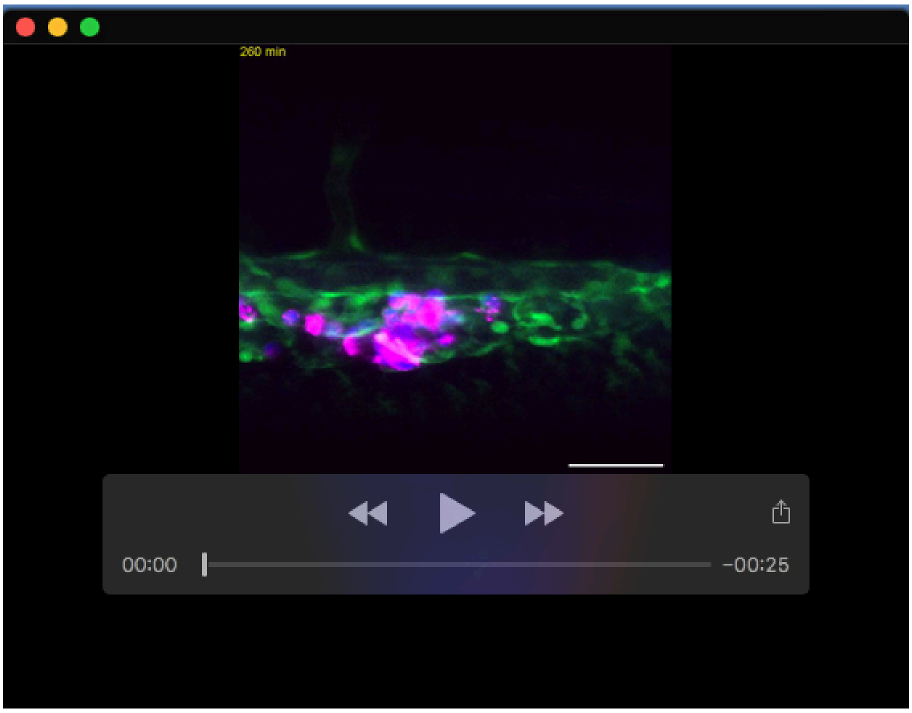


**Movie 2.** Confocal timelapse of ZMEL cancer cells (magenta, blue nuclei) extravasating from the tail vessels (green) of a 2 dpf larva. Scale bar = 50 µm.

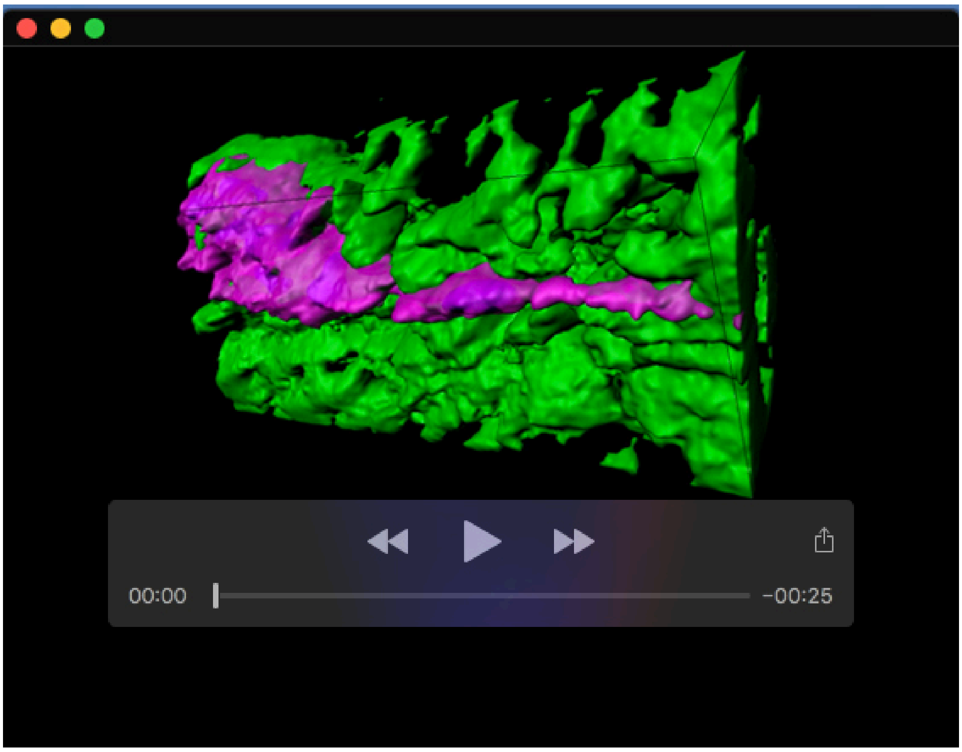


**Movie 3.** Imaris 3D rendering showing 360° rotation of ZMEL cancer cells (magenta, blue nuclei) after exiting the vasculature (green) of a 2 dpf larva.

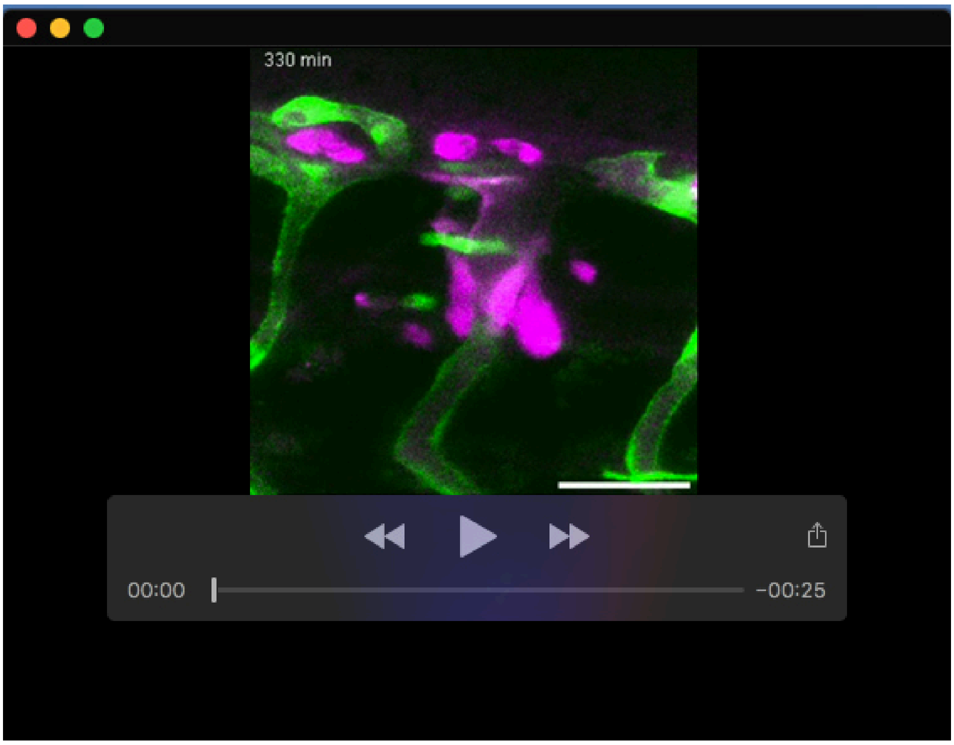


**Movie 4.** Confocal timelapse imaging of a human PC3 prostate cancer cell (magenta) integrating into the vasculature (green) of a 3 dpf larva, after ablation of an intersegmental vessel.

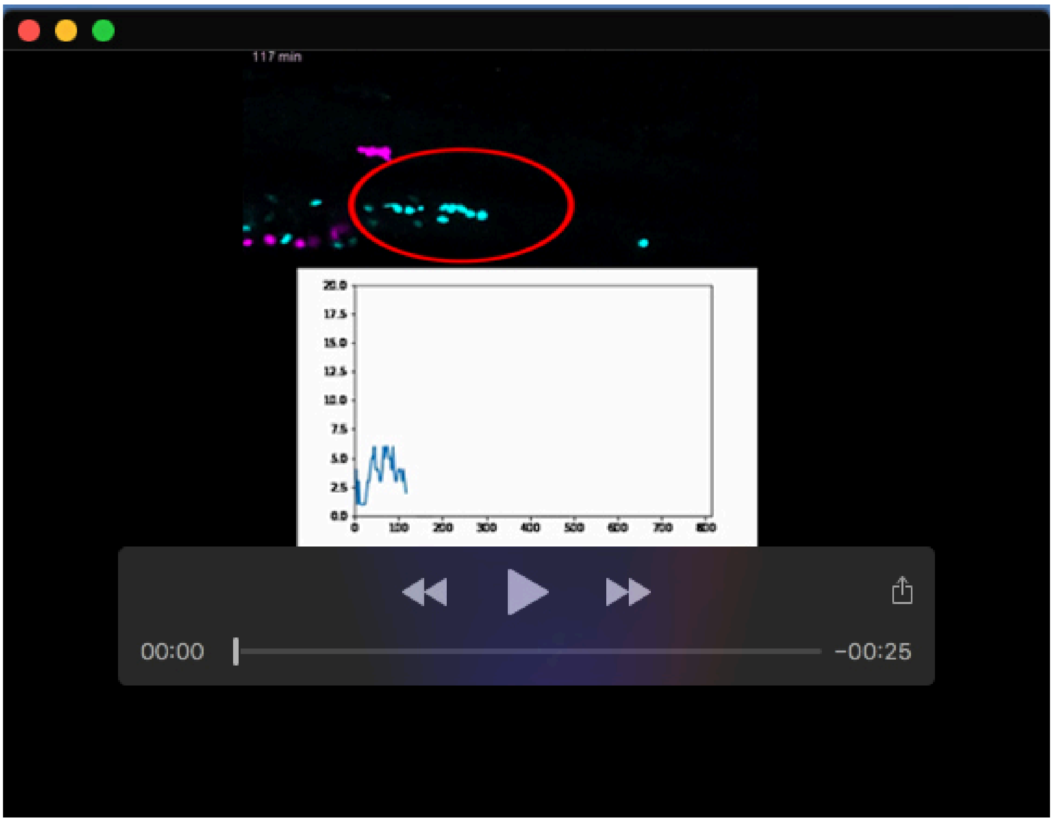


**Movie 5.** Timelapse of neutrophil (magenta) recruitment after laser-induction of a thrombus (red circle, cyan cells). An animated graph shows the neutrophil count in the region throughout imaging, with a red line indicating the time at which the thrombocytic plug is resolved.

Journal of Cell Science • Supplementary information
