# Supplementary material for: Live-imaging studies reveal how microclots and the associated inflammatory response enhance cancer cell extravasation
Source: J Cell Sci. 2023 Sep 28;136(18):jcs261225. doi: 10.1242/jcs.261225 (PMC10561694; doi:10.1242/jcs.261225)
Supplement: Supplementary file 1 [file joces-136-261225_review_history.pdf]

Live-imaging studies reveal how microclots and the associated inflammatory response enhance cancer cell extravasation

Juma Ward and Paul Martin
DOI: 10.1242/jcs.261225

Editor: Caroline Hill

Review timeline
Original submission: 11 April 2023
Editorial decision: 23 May 2023
First revision received: 24 August 2023
Accepted: 31 August 2023

Original submission

First decision letter

MS ID#: JOCES/2023/261225

MS TITLE: Live imaging studies in zebrafish reveal how microclots and the associated inflammatory response enhance cancer cell extravasation

AUTHORS: Juma Ward and Paul Martin
ARTICLE TYPE: Short Report

We have now reached a decision on the above manuscript.

To see the reviewers' reports and a copy of this decision letter, please go to: https://submit-jcs.biologists.org and click on the 'Manuscripts with Decisions' queue in the Author Area. (Corresponding author only has access to reviews.)

As you will see, the reviewers raise a number of criticisms that prevent me from accepting the paper at this stage. They suggest, however, that a revised version might prove acceptable, if you can address their concerns. If you think that you can deal satisfactorily with the criticisms on revision, I would be very pleased to see a revised manuscript. A couple of the issues will require new experiments, but many of them will only require text and Figure reworking.

Please ensure that you clearly highlight all changes made in the revised manuscript. Please avoid using 'Tracked changes' in Word files as these are lost in PDF conversion.

I should be grateful if you would also provide a point-by-point response detailing how you have dealt with the points raised by the reviewers in the 'Response to Reviewers' box. Please attend to all of the reviewers' comments. If you do not agree with any of their criticisms or suggestions please explain clearly why this is so.

Reviewer 1

Advance summary and potential significance to field

In this article, researchers evaluated the relationship between platelets, endothelial cells, and inflammatory cells in a zebrafish model. To date, studies of coagulation processes and their

© 2023. Published by The Company of Biologists under the terms of the Creative Commons Attribution License (https://creativecommons.org/licenses/by/4.0/).

interactions with tumorigenesis have been conducted mainly in rodents. In this study, researchers use the zebrafish model to study cell-cell interactions in the tumor niche. The choice of the model for this research seems most reasonable. Thanks to the translucency of its body, the model makes it possible to follow the changes taking place under in vivo conditions. This article not only confirms the antitumor effect of warfarin (as previously reported in other studies), but examines its effect on components related to the tumor environment. The involvement of coagulation processes on the tumor process was also confirmed, and their behavior and roles on tumor extravasation were revealed.In addition, this study contributes to a better understanding of tumor biology at the cellular level. The role of selected compotents involved in immune responses to tumor metastasis was also investigated. In addition, the researchers identified the role of TNFalpha on tumor cells, which also expands the knowledge regarding this cytokine, as its role in the process of tumorigenesis is the topic of numerous scientific discussions. Understanding the important tumor microenvironment at the cellular level, is important in terms of selecting appropriate therapeutic targets.

Comments for the author

The researchers do not explain why exactly they chose the PC3 prostate cancer cell line for this study. There is also no information on where the cell line was purchased from. In the methodology, the researchers describe the use of a breast cancer line (MDA-MB231-RFP). However, it does not appear in any other part of the manuscript.

In the methodology, some reagents have supplier information and some do not. This should be unified.

100% DMSO? Maximal purity of DMSO is >= 99.99%. I have no further objections to the methodology. The injection was conducted in an appropriate place. For gene knockdown, it would have been better to use the CRISPR/Cas9 method, but using morpholino is fine, due to the fact that the researchers worked on larvae up to 5 days old. However, for the future, it would be worth considering this method in other studies.

Figures:
Figures could be more readable. In particular, figures 1N, 3B, 4H, 3K, 3M. In addition, the text in the figures is of different sizes, e.g. 3D , 3E and 3F, 3G. Lettering designations for the figures would be good to arrange at one height. The figures would need to be organized and unified. It would also be good to indicate where statistical significance was, not just give the p-value alone.

<u>Reviewer 2</u>

Advance summary and potential significance to field

This work investigates the dynamic interaction between platelets, endothelial cells, inflammatory cells, and engrafted human and zebrafish cancer cells by live imaging studies in translucent zebrafish larvae. The authors show how clotting (and clot resolution) act as foci and trigger for extravasation. In addition inflammatory macrophage inhibits cancer cell extravasation. Overall, the work is highly significant in understanding cancer metastasis and related cancer therapy.

Comments for the author

1. Whereas the results are interesting, the exact number of repeats and the number of animals used in each experiment are not specified in the result or figure legend section. This information would be necessary to ensure the results are reproducible and unbiased.
2. Including timelapse or movies of the extravasation process will provide additional information about the extravasation process in addition to the snapshots.
3. The results in Figure 2E-H suggest possible cancer cell integration into the damaged vasculature. A similar CLEM study on the damaged region will allow better visualization of cell-cell interactions in this process. The thrombocytes are likely recruited to the damage sites in addition to macrophages. Is clotting necessary for this integration?

© 2023. Published by The Company of Biologists under the terms of the Creative Commons Attribution License (https://creativecommons.org/licenses/by/4.0/).

4. The reviewer had difficulty understanding the statistics in Fig.3K. How are the groups defined?
5. The fga MO should be validated. The knockdown efficiency can be checked with RT-PCR of the fga transcripts. The author can rescue the morphant phenotype with fibrin injection to ensure the fibrin loss drives the phenotype and rule out off-target effects.
6. Results in Figure 4I-L support the role of inflammatory stimuli in triggering TNFalpha+ cells and limiting cancer cell extravasation. However, this experimental design does not include clot formation. Therefore the last sentence in the abstract “TNFα+ve phenotype in macrophages is inhibitory to clot mediated extravasation of cancer cells.” is an overstatement. First of all, the experiment is not performed under clot-forming conditions. In addition, systemic R848 administration is not macrophage-specific. Factors in addition to macrophages can contribute to this result as well.
7. Overall, the result section should include more details of the experimental procedure to facilitate readers' understanding of the experiments and outcomes.
8. The discussion section should be expanded to have a comprehensive discussion of how these new results integrate with the literature and possible underlying mechanisms of how clots and phagocytes drive cancer cell extravasation.

First revision

Author response to reviewers' comments

Reviewer 1 Comments for the Author...

The researchers do not explain why exactly they chose the PC3 prostate cancer cell line for this study. There is also no information on where the cell line was purchased from. In the methodology, the researchers describe the use of a breast cancer line (MDA-MB231- RFP). However, it does not appear in any other part of the manuscript.

PC3 prostate cancer cells and MDA-MB-231 breast cancer cells were chosen as examples of highly metastatic human cancer cell lines. MDA-MB-231 were used in Figure 1 for live- imaging of human cancer cell extravasation; please excuse this omission from the legend, which we have now corrected. And we have added additional details regarding the sources of each of these cell lines to the Methods section (page 9, paragraph 3 and 4)

In the methodology, some reagents have supplier information and some do not. This should be unified.

Done.

100% DMSO? Maximal purity of DMSO is >= 99.99%.

Indeed, our DMSO was only >=99.9%; we have amended this in the text.

I have no further objections to the methodology. The injection was conducted in an appropriate place. For gene knockdown, it would have been better to use the CRISPR/Cas9 method, but using morpholino is fine, due to the fact that the researchers worked on larvae up to 5 days old. However, for the future, it would be worth considering this method in other studies.

Agreed, for future experiments, CRISPR/Cas9 knockout might be appropriate. However, in this instance we think the morpholino knockdown is better; a CRISPR/Cas9 knockout zebrafish for fga has been previously published (Fish *et al.* 2021 *Int J Mol Sci*) but was reported as having a less extreme haemostatic phenotype by comparison to the morpholino knockdown, showing no spontaneous bleeding.

Figures:
Figures could be more readable. In particular, figures 1N, 3B, 4H, 3K, 3M. In addition, the text

© 2023. Published by The Company of Biologists under the terms of the Creative Commons Attribution License (https://creativecommons.org/licenses/by/4.0/).

in the figures is of different sizes, e.g. 3D , 3E and 3F, 3G. Lettering designations for the figures would be good to arrange at one height.
The figures would need to be organized and unified.
It would also be good to indicate where statistical significance was, not just give the p- value alone.

Thanks. We have now attended to these concerns regarding figure layout, and have added asterisks to indicate statistical significance, where appropriate.

Reviewer 2 Comments for the Author...

1. Whereas the results are interesting, the exact number of repeats and the number of animals used in each experiment are not specified in the result or figure legend section. This information would be necessary to ensure the results are reproducible and unbiased.

Sorry; we've now added this information to the Legends within the paper.

2. Including timelapse or movies of the extravasation process will provide additional information about the extravasation process in addition to the snapshots.

Movies of the extravasation process (from which the still images were taken) have now been included as part of the supplemental data (Supp Movies 1 and 2).

3. The results in Figure 2E-H suggest possible cancer cell integration into the damaged vasculature. A similar CLEM study on the damaged region will allow better visualization of cell-cell interactions in this process. The thrombocytes are likely recruited to the damage sites in addition to macrophages. Is clotting necessary for this integration?

While CLEM of this possible vasculogenic mimicry would certainly provide additional clarity and might reveal interesting details, a new CLEM study would be a major enterprise for us which we guestimate would take more than 6 months of work. Moreover, the first author, JW, has moved on from the Martin lab, and so this would further complicate the process.

Our study is suggestive, rather than definitive, that clotting is key to the vascular mimicry. Tumour associated macrophages are drivers of vasculogenic mimicry (Liu *et al*. 2022, *Cell Death Dis* - we now cite this paper in our text (page 5, paragraph 2), and our own results have shown an increased recruitment of macrophages in the presence of activated thrombocytes.

4. The reviewer had difficulty understanding the statistics in Fig.3K. How are the groups defined?

The data in Fig 3K was a comparison between larvae with a clot and larvae with a control laser wound that would not cause a clot. This was analysed by two-way ANOVA with post-hoc testing, showing a significant difference between the groups at the peak of neutrophil influx, but not before or after this point. We have now clarified this in the text (page 6 paragraph 3), and in the figure.

5. The fga MO should be validated. The knockdown efficiency can be checked with RT-PCR of the fga transcripts. The author can rescue the morphant phenotype with fibrin injection to ensure the fibrin loss drives the phenotype and rule out off-target effects.

This MO has been validated previously (Vilar *et al*. 2021 *Thromb Haem*), and we have now added our own PCR data (Fig 4 H') to show how efficiently it knocks down fibrinogen sub unit. Unfortunately, we are not in a position to do a rescue experiment because we ran down all of our ZMEL-1 cells and have been unable procure fresh stocks from another European source. Richard White, the primary source is currently moving labs from US to Oxford. Hope you understand.

6. Results in Figure 4I-L support the role of inflammatory stimuli in

© 2023. Published by The Company of Biologists under the terms of the Creative Commons Attribution License (https://creativecommons.org/licenses/by/4.0/).

triggering TNFalpha+ cells and limiting cancer cell extravasation. However, this experimental design does not include clot formation. Therefore the last sentence in the abstract "TNFα+ve phenotype in macrophages is inhibitory to clot mediated extravasation of cancer cells." is an overstatement. First of all, the experiment is not performed under clot-forming conditions. In addition, systemic R848 administration is not macrophage-specific. Factors in addition to macrophages can contribute to this result as well.

We have shown that the presence of circulating tumour cells appears to induce clot- forming conditions in the absence of any other stimulation, through injection of exogenous fibrinogen, which is converted to fibrin. However, it's true, the TNFα phenotype induced by R848 is not restricted to macrophages, so we agree, we need to modify the last sentence of our Abstract, which we have now done.

7. Overall, the result section should include more details of the experimental procedure to facilitate readers' understanding of the experiments and outcomes.

We have added a little more detail here, particularly in the case of drug treatments and our tracking protocol, but in the earlier submission (and this revision too), we have been somewhat mindful of word limit constraints.

8. The discussion section should be expanded to have a comprehensive discussion of how these new results integrate with the literature and possible underlying mechanisms of how clots and phagocytes drive cancer cell extravasation.

Ditto; but we have now included some additional discussion/speculation re the role of fibrin in the pre-metastatic niche (page 8, paragraph 3).

<u>Second decision letter</u>

MS ID#: JOCES/2023/261225

MS TITLE: Live imaging studies reveal how microclots and the associated inflammatory response enhance cancer cell extravasation

AUTHORS: Juma Ward and Paul Martin
ARTICLE TYPE: Short Report

I am happy to tell you that your manuscript has been accepted for publication in Journal of Cell Science, pending standard ethics checks.

© 2023. Published by The Company of Biologists under the terms of the Creative Commons Attribution License (https://creativecommons.org/licenses/by/4.0/).
